# Altered peripheral immune profiles in treatment-resistant depression: response to ketamine and prediction of treatment outcome

**DOI:** 10.1038/tp.2017.31

**Published:** 2017-03-21

**Authors:** D D Kiraly, S R Horn, N T Van Dam, S Costi, J Schwartz, S Kim-Schulze, M Patel, G E Hodes, S J Russo, M Merad, D V Iosifescu, D S Charney, J W Murrough

**Affiliations:** 1 Mood and Anxiety Disorders Program, Department of Psychiatry, Icahn School of Medicine at Mount Sinai, New York, NY, USA; 2 Fishberg Department of Neuroscience, Friedman Brain Institute, Icahn School of Medicine at Mount Sinai, New York, NY, USA; 3 Seaver Autism Center for Research and Treatment, Icahn School of Medicine at Mount Sinai, New York, NY, USA; 4 The Immunology Institute, Human Immune Monitoring Core, Icahn School of Medicine at Mount Sinai, New York, NY, USA; 5 Department of Pharmacology and Systems Therapeutics, Icahn School of Medicine at Mount Sinai, New York, NY, USA

## Abstract

A subset of patients with depression have elevated levels of inflammatory cytokines, and some studies demonstrate interaction between inflammatory factors and treatment outcome. However, most studies focus on only a narrow subset of factors in a patient sample. In the current study, we analyzed broad immune profiles in blood from patients with treatment-resistant depression (TRD) at baseline and following treatment with the glutamate modulator ketamine. Serum was analyzed from 26 healthy control and 33 actively depressed TRD patients free of antidepressant medication, and matched for age, sex and body mass index. All subjects provided baseline blood samples, and TRD subjects had additional blood draw at 4 and 24 h following intravenous infusion of ketamine (0.5 mg kg^−1^). Samples underwent multiplex analysis of 41 cytokines, chemokines and growth factors using quantitative immunoassay technology. Our *a priori* hypothesis was that TRD patients would show elevations in canonical pro-inflammatory cytokines; analyses demonstrated significant elevation of the pro-inflammatory cytokine interleukin-6. Further exploratory analyses revealed significant regulation of four additional soluble factors in patients with TRD. Several cytokines showed transient changes in level after ketamine, but none correlated with treatment response. Low pretreatment levels of fibroblast growth factor 2 were associated with ketamine treatment response. In sum, we found that patients with TRD demonstrate a unique pattern of increased inflammatory mediators, chemokines and colony-stimulating factors, providing support for the immune hypothesis of TRD. These patterns suggest novel treatment targets for the subset of patients with TRD who evidence dysregulated immune functioning.

## Introduction

Major depressive disorder (MDD) is a debilitating condition that can have profound effects on both the mind and the body of individuals who suffer from the disorder. Research into novel, more effective treatments for depression has been hampered by an incomplete understanding of underlying pathophysiology.^1^ At the present time, all Food and Drug Administration-approved treatments for depression alter levels of monoamine neurotransmitters. However, there is a large subset of patients with MDD who do not show adequate response to these drugs—these patients are generally characterized as having treatment-resistant depression (TRD). Although the mechanisms of treatment resistance are not well understood, TRD patients represent a large fraction of patients with MDD^2^—making the understanding of pathophysiology and alternative treatment strategies a critical research aim.

Numerous studies have measured alterations in cytokines in the blood and cerebrospinal fluid (CSF) of patients with major depression,^3, 4, 5, 6^ and elevated levels of cytokines in adolescence have been associated with increased susceptibility to depression in adulthood.^7^ Some studies point to a role for increased inflammation specifically in patients with TRD.^4, 8, 9^ Although these findings have been consistently reported, there is considerable variability between individuals, and anti-inflammatory treatments for depression in patients not pre-screened for elevated inflammatory markers have thus far only limited clinical efficacy.^10, 11^ This has led to the hypothesis that there is a subset of MDD cases, enriched in TRD populations, driven by inflammatory processes, whereby anti-inflammatory treatments have the potential to be viable alternative treatment strategies.^3, 4^

A wealth of recent evidence has also demonstrated alterations in signaling and metabolism of glutamate in patients with MDD.^12, 13^ The importance of glutamate in depression has been particularly highlighted by the emergence of the N-methyl-D-aspartate receptor antagonist, ketamine, as a rapidly acting antidepressant.^14, 15, 16^ Of particular interest to us, there are a number of studies demonstrating that ketamine also has anti-inflammatory properties. Multiple clinical and pre-clinical studies have shown evidence for reduced inflammation with ketamine,^17, 18^ and in animal models ketamine is able to reverse inflammation-induced depression and decrease brain levels of inflammatory cytokines.^19, 20^ The effect of ketamine on inflammation in depressed patients is somewhat mixed in the literature with one small study suggesting that ketamine reduced serum interleukin (IL)-6 in a manner that correlated with treatment response,^21^ and another showing ketamine causing a transient increase in IL-6 in a manner that did not correlate with response.^22^ Mounting evidence suggests that changes in inflammatory signaling influence glutamatergic transmission in the brain.^3, 4, 23, 24^ In animal models, ketamine reversal of inflammation-induced depressive-like behavior is blocked by the inhibition of glutamatergic transmission.^20^ Human imaging studies have shown that altered inflammation can change glutamate levels in the frontal cortex^25^ and basal ganglia,^26^ and that patients with increased inflammation have decreased connectivity in corticostriatal reward circuits.^27^ Given the links between glutamate, inflammation and depression, ketamine may modulate inflammatory signaling in ways that contribute to its antidepressant efficacy.

The current study examines a broad panel of inflammatory mediators in TRD patients compared with healthy controls (HCs). Although there is a large literature demonstrating changes in inflammatory and immune mediators associated with symptomatic depression,^5, 7, 11, 28, 29, 30^ most of these studies have focused on only a few analytes. For this study, we performed an analysis with the *a priori* hypothesis that canonical inflammatory proteins (that is, IL-6, IL-1α/β and TNF-α) would be elevated in TRD, and then we performed hypothesis-generating exploratory analysis on a wide range of other factors. The current data set provides a comprehensive within-subject inflammatory profile of TRD patients before and after treatment with ketamine. These data can be used to develop further hypotheses about the role of inflammation in depression, and provide a foundation for biomarker research aimed at predicting treatment response to ketamine.

## Materials and methods

### Study design and patients

Male and female adults with TRD and HC volunteers were recruited for the current study through an outpatient psychiatric research program at Icahn School of Medicine at Mount Sinai. Inclusion criteria for the TRD group included having a primary diagnosis of MDD determined using the Structured Clinical Interview for Diagnostic and Statistical Manual of Mental Disorders, 4th edition,^31^ being in a current major depressive episode of at least moderate severity, and having a history of non-response to at least two therapeutic trials of an antidepressant according to the criteria of the Antidepressant Treatment History Form.^32^ Depression severity at the time of ketamine infusion was determined using the Montgomery–Åsberg Depression Rating Scale.^33^ All participants were free of all antidepressant medications for at least 2 weeks prior to starting the study protocol. No participants in this study had recently been on fluoxetine, which would have necessitated a longer antidepressant-free period. TRD patients were excluded if they had a lifetime history of a psychotic disorder or bipolar disorder, alcohol or substance abuse in the previous 6 months, unstable medical illness, or active nicotine use. Within the TRD group, three patients had stable hypertension, two hyperlipidemia and one with mild type II diabetes. Prohibited medications included antidepressants and other psychotropic agents and all medications that could potentially affect the immune system (for example, nonsteroidal anti-inflammatory drugs). Participants were free of current substances of abuse as determined by a urine toxicology test at the time of screening. Healthy volunteers were free of lifetime psychiatric illness or significant medical conditions. All participants were free of active infections or systemic illness as confirmed by medical history and a complete review of systems.

Individuals with TRD meeting the above criteria were eligible for the current study if they were also enrolled in a concurrent clinical trial of ketamine at Mount Sinai (ClinicalTrials.gov IDs: NCT01880593, NCT00768430 and NCT00548964). A concurrently enrolled HC group was selected using frequency matching on the variables of age, sex and body mass index, as these variables are know to affect inflammatory markers. The Program for the Protection of Human Subjects at Icahn School of Medicine at Mount Sinai approved the study. After complete description of the study to potential participants, written informed consent was obtained prior to the conduct of any study procedures. Sample size was determined using G*power software (Düsseldorf, Germany) to detect a moderate effect size with 80% power and an *α* of 0.05

### Sample collection and ketamine infusion procedures

All blood samples were obtained via antecubetal venous collection using standard techniques. Baseline samples from HC and TRD subjects were drawn between 0800 and 0900 hours after a period of overnight fasting (>8 h). TRD participants provided a blood sample (see below) on the morning prior to receiving a single intravenous infusion of ketamine (0.5 mg kg^−1^) in a clinical research unit setting. As in previous studies,^16^ change in depressive severity from baseline to +24 h following ketamine infusion represented the primary clinical outcome. Subsequent samples were obtained from the same patients a +4 and +24 h following the treatment. Detailed study drug infusion methods were presented previously.^16, 34^

### Multiplex analysis

Measurement of cytokines, chemokines and growth factors was performed on serum samples isolated as above. Multiplex analysis was performed using the commercially available human cytokine/chemokine magnetic bead panel (Millipore, Billerica, MA, USA, HCYTMAG-60 K-PX38 and HNDG3MAG-36 K for brain-derived neurotrophic factor) on a Luminex 200 multiplex immunoassay system at the Human Immune Monitoring Core at Mount Sinai. Samples were run on three plates with groups evenly divided between the three to account for any inter-plate variability. Assays were quality control checked for fit to a standard curve and for coefficient of variation (data available as Supplementary Table S1). In addition, levels of IL-6 from this assay were correlated with enzyme-linked immunosorbent assay analysis of the same samples to ensure that there was strong correlation between the two methods—this correlation is presented as Supplementary Figure S1. Four samples (two HCs and two TRD) had aberrant values on every analyte and were thus excluded from further analyses.

### Data analysis

Data were analyzed and graphed using SPSS (v21, OSX, IBM, Armonk, NY, USA) and GraphPad Prism (v7, GraphPad, La Jolla, CA, USA). For all analytes, any samples that were below the detectable limit of the assay were included as half of the lower limit of detection.^22^ Thirteen analytes (IL-2, IL-3, IL-4, IL-5, IL-9, IL-10, IL-12p40, IL-12p70, IFNα, FLT3L, MCP-3, TGF-α and TNF-β) had >50% of values below the limit of detection for the assay. Data were log-transformed prior to analysis. Baseline group differences were examined using linear regression; analyses were conducted both with and without the inclusion of age, sex, body mass index and race as covariates. Changes in cytokine levels at 4 and 24 h were performed via paired *t*-tests. Analysis of serum proteins as predictors of ketamine treatment response was performed initially as an independent *t*-test and then confirmed via logistic regression analysis accounting for covariates as above. On the basis of our *a priori* hypothesis that inflammatory cytokines would be elevated in TRD patients, we corrected for multiple comparisons only in that analysis. The remainder of the analyses statistical correction for multiple comparisons was not performed. Unless otherwise stated, all *P*-values in the main text are uncorrected and unadjusted for covariates. Adjustment for covariates did not change statistical significance in any case and adjusted values are listed in Supplementary Table S2.

## Results

### Patient characteristics

Thirty-three patients with TRD and 26 healthy volunteers completed all study procedures and are included in the analyses. The sociodemographic and clinical characteristics of the patients are presented in Table 1. Although analyses were performed on log-transformed data, raw pg ml^−1^ values for all inflammatory and exploratory cytokines are included in Table 2.

### Inflammatory cytokines in TRD and before and after ketamine

**Figure 1:**
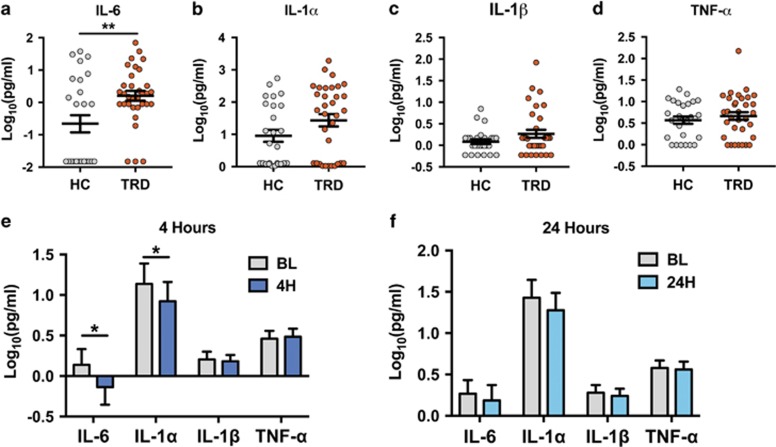
Differences in inflammatory cytokines in TRD patients before and after treatment with ketamine. (**a–d**) When compared to healthy controls (HC) treatment-resistant depression (TRD) patients exhibited significantly elevated levels of interleukin (IL)-6 (*P*=0.004), but not other inflammatory markers. (**e**) Levels of IL-6 and IL-1α significantly decrease in the serum of patients 4 h after treatment with 0.5 mg kg^−1^ intravenous ketamine—*P*<0.05 for both. (**f**) Twenty-four hours after ketamine infusion, the levels of all inflammatory cytokines were no longer significantly different from baseline levels (*P<0.05; **P<0.01; error bars represent s.e.m.).

When comparing HC subjects to medication-free TRD subjects at baseline, TRD patients demonstrated significantly elevated levels of IL-6 after Bonferroni correction for multiple comparisons (Figure 1a—raw *P*=0.004; corrected *P*=0.01). Levels of IL-1α, IL-1β and TNF-α were not significantly different between groups (Figures 1b–d). On examination of cytokine levels 4 h post treatment with ketamine, IL-6 and IL-1α showed modest but statistically significant decreases from baseline (*P*<0.05; *t*=2.369 and *P*<0.05, *t*=2.149, respectively; Figure 1e). By 24 h, there were no longer any significant differences in inflammatory cytokine levels from baseline (Figure 1f). Changes in cytokine levels were not associated with treatment response to ketamine at either time point.

### Exploration of altered cytokines, chemokines and growth factors in TRD patients

**Figure 2:**
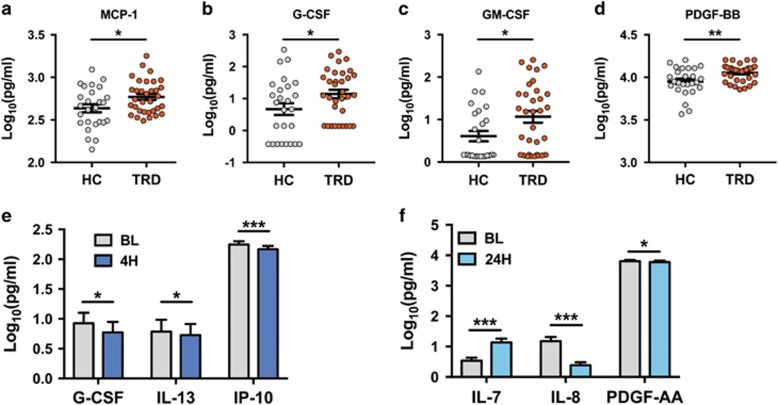
Significant differences in exploratory serum proteins. (**a–d**) Analysis of baseline serum from healthy control (HC) and treatment-resistant depression (TRD) subjects revealed elevated MCP-1 (*P*=0.02), G-CSF (*P*=0.037), GM-CSF (*P*=0.02) and PDGF-BB (*P*=0.005) in patients with TRD. (**e**) At 4 h post ketamine treatment, levels of G-CSF (*P*=0.038), interleukin (IL)-13 (*P*=0.038) and IP-10 (*P*<0.0001) had decreased from their baseline values. (**f**) At 24 h after ketamine treatment, levels of IL-7 were increased (*P*<0.0001), IL-8 was decreased (*P*<0.0001) and PDGF-AA decreased (*P*=0.024). (*P<0.05; **P<0.01; ***P<0.005; error bars represent s.e.m.). CSF, cerebrospinal fluid.

Data from the remaining exploratory analytes were compared between HC and TRD subjects (Table 2; Figure 2). This analysis yielded significant differences between HC and TRD for four different analytes—all of which were elevated in patients with TRD (Figures 2a–d). Among them was the chemokine MCP-1 (*P*=0.02) and several colony-stimulating and growth factors—G-CSF (*P*=0.037), GM-CSF (*P*=0.02) and PDGF-BB (*P*=0.005). Notably, brain-derived neurotrophic factor did not change with ketamine treatment, and was not a predictor of treatment response at any time point (Supplementary Figure S2).

As with the inflammatory cytokines examined in Figure 1, we also analyzed this panel of exploratory markers to determine whether any of them were influenced by the treatment with ketamine. Of the 37 examined, only three showed modest changes after ketamine treatment (Figure 2e). G-CSF (*P*=0.038), IL-13 (*P*=0.038) and IP-10 (*P*<0.0001) all showed statistically significant decreases 4 h after treatment, but none were significantly different from baseline level at the 24-h time point. There were three other analytes that showed very modest, but statistically significant changes at 24 h; levels of IL-7 were increased (*P*<0.0001), IL-8 was decreased (*P*<0.0001) and PDGF-AA decreased (*P*=0.024) at the 24-h time point (Figure 2f), although none were altered at the 4-h time point. As with the cytokines described in Figure 1, none of the alterations in these exploratory analytes correlated with antidepressant treatment response.

### Serum fibroblast growth factor 2 as a predictor of ketamine treatment response

**Figure 3:**
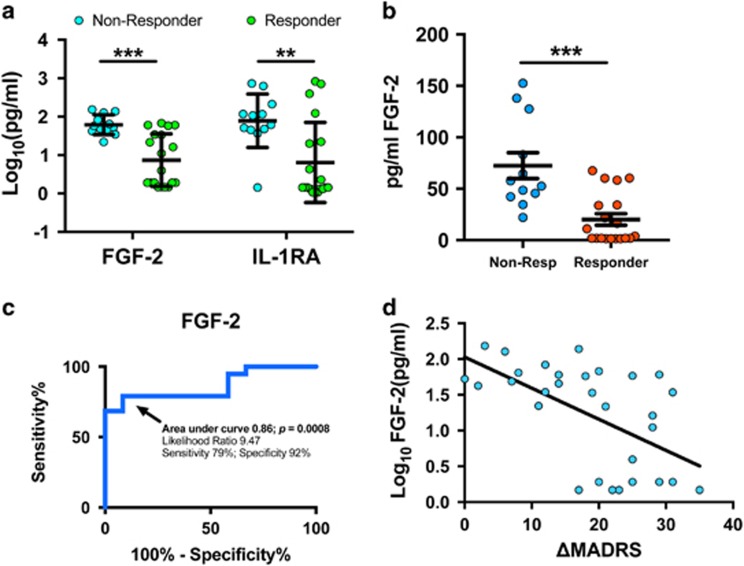
Low serum levels of FGF-2 predict treatment response to ketamine. Analysis of serum levels of all factors was examined in all patients by treatment response (⩾50% reduction in MADRS at 24 h) versus non-response. (**a**) These analyses showed that lower serum levels of FGF-2 (*P*=0.0001) and IL-1ra (*P*=0.0035) were seen in treatment responders with a more robust and reliable difference in FGF-2. (**b**) Non-transformed pg ml^−1^ values for serum FGF-2 are presented. (**c**) Receiver-operating characteristic analyses of FGF-2 in both groups showed that those patients with a serum level below 34 pg ml^−1^ had a 9.47 likelihood ratio of treatment response with 79% sensitivity and 92% specificity (area under curve 0.86; *P*=0.0008). (**d**) Correlation between baseline levels of FGF-2 and the change in MADRS score in the TRD group at 24 h following ketamine are shown. Positive numbers on the *X* axis denote extent of improvement in Montgomery–Åsberg Depression Rating Scale (MADRS; Pearson's *r*=−0.565; *P*=0.0009). (**P<0.01; ***P<0.005; error bars represent s.e.m.). FGF-2, fibroblast growth factor 2; IL, interleukin; TRD, treatment-resistant depression.

In an attempt to find baseline predictors of therapeutic response to ketamine, we analyzed levels of all factors in patients who did and did not show adequate response to a single infusion to ketamine (24-h response rate was 60% for this cohort). From this analysis, we found that patients who went on to respond to ketamine had significantly lower pretreatment levels of fibroblast growth factor 2 (FGF-2) (*P*=0.0001 unadjusted; *P*=0.03 in a model correcting for covariates; Figure 3a) and IL-1ra (*P*=0.0035 unadjusted; *P*=0.033 in a model correcting for covariates). Given that FGF-2 was more consistently low in treatment responders, we performed further analysis of its viability as a biomarker of treatment response. Non-transformed pg ml^−1^ values are presented in Figure 3b. Receiver-operating characteristic analysis demonstrated that using a cutoff of <34 pg ml^−1^ (log_10_-transformed value of 1.536) the likelihood ratio of treatment response was 9.47 with a sensitivity of 79% and a specificity of 92% (Figure 3c; *P*=0.0008). These findings are further graphically demonstrated in Figure 3d, showing the correlation of baseline FGF-2 with the change in Montgomery–Åsberg Depression Rating Scale score at 24 h (Pearson's *r*=−0.565; *P*=0.0009).

## Discussion

In this study, we examined peripheral immune profiles in medication-free patients with TRD before and after treatment with ketamine. In performing a broad characterization of inflammatory signaling pathways, we found a unique serum profile not previously described within a group of TRD patients. Although some findings, such as elevated IL-6, were anticipated and are consistent with previous observations from our group and others,^30, 35^ we also found a pattern of upregulated chemokines, colony-stimulating factors and growth factors in TRD. Consistent with previous reports, these data indicate that there is a subset of TRD patients with increased inflammatory markers, and this may be useful in prediction of treatment response.^4, 10^ We identified low levels of serum FGF-2 as a specific predictor of ketamine treatment response. Taken together, these findings bolster the connection between alteration in peripheral inflammation and TRD, and provide information that may be utilized for future translational research and drug discovery efforts.

### Inflammation and treatment response

In our examination of pro-inflammatory cytokines in TRD patients, we found IL-6 to be robustly and significantly elevated in the depressed population (Figure 1). Both forms of IL-1 showed trends towards increases, but had high variability. This is generally consistent with multiple previous studies and meta-analyses.^5, 30^ In the TRD patients, ketamine decreased serum levels of IL-6 and IL-1α 4 h after the infusion, but levels had returned to baseline by 24 h after the infusion. Importantly, the decrease in levels of IL-6 was modest and did not correlate with clinical response to ketamine treatment. Anti-inflammatory properties of ketamine have been previously demonstrated,^17, 18^ and we would posit that our results are in agreement with anti-inflammatory effects of ketamine, but they do not support an anti-inflammatory mechanism in the resolution of depressive symptoms. It is important to note that these changes in peripheral cytokines are not necessarily reflective of the inflammatory state of the central nervous system (CNS), and it is possible that ketamine could be affecting levels of cytokines centrally.

### Exploratory cytokines

There are several interesting possibilities that arise from the significantly upregulated factors in our TRD population. Among the regulated exploratory markers, we found two colony-stimulating factors, G-CSF and GM-CSF, which are well characterized for their ability to mobilize leukocytes from the bone marrow.^36^ In addition, we also observed upregulation of the chemotactic cytokine MCP-1 (CCL2; Figure 2). This pattern of analytes suggests a model in which a subset of patients may have increased mobilization of monocytes from the bone marrow, and increased chemotactic factors drawing them to target organs—including the brain. Although our data cannot provide mechanistic insight into this hypothesis, there is a growing body of evidence that during periods of stress, activated monocytes are recruited into the brain parenchyma and lead to a pro-inflammatory CNS environment.^3, 23^ MCP-1, in particular, has been implicated in recruiting activated monocytes from the peripheral circulation,^23, 37^ and has been shown to be upregulated in the brains of depressed suicide completers.^38^ Interestingly, rodent social stress models show that leukocyte-specific knockout of the receptor for MCP-1 prevents stress-induced monocyte recruitment into the brain as well as the maladaptive behavioral effects of stress,^39^ and that these leukocytes that are recruited in an MCP-1-dependent manner increase pro-inflammatory signaling in the brain through IL-1 signaling pathways.^40^ Although we lack direct evidence for monocyte recruitment into the human CNS in TRD subjects, further exploration of this hypothesis is warranted.

### FGF-2 as a predictor of ketamine treatment response

Development of biomarkers that can predict treatment response is of paramount importance in psychiatry. Although response rates to ketamine tend to be >50%,^41^ a more accurate pre-test prediction would likely be of clinical utility. Previous studies have suggested baseline delta sleep ratio,^42^ CNS glutamate levels^43^ or cognitive function^34^ may predict treatment response to ketamine. However, these tests are expensive and labor-intensive in their own regard—somewhat decreasing their possible utility as screening tools. Other studies have suggested that family history of alcoholism^44^ or an anxious subtype of depression^45^ may be predictive of better treatment response. With respect to peripheral markers of ketamine treatment response, one previous study demonstrated that higher serum levels of the synaptic protein Shank3 correlated with improvement following ketamine administration in bipolar depression^46^; a second study found that lower levels of adiponectin, an anti-inflammatory protein, correlated with treatment response in MDD and bipolar depression.^47^ In our study, we found that low levels of FGF-2 (<34 pg ml^−1^) were predictive of ketamine treatment response (92% specific; Figure 3). Interestingly, FGF-2 has received considerable attention for its role in depression. Studies have shown reduced levels of FGF-2 in serum from patients with MDD,^48^ and post-mortem analysis has shown decreased levels of FGF-2 and related transcripts in the frontal cortices of MDD patients.^49^ In rodents, FGF-2 is required for the effect of antidepressant medications, and FGF-2 has been shown to be pro-neurogenic and pro-gliogenic.^50^ A recent study showed that loss of FGF-2-producing NG2 glia from the frontal cortex of mice leads to depressive-like behaviors and dysregulated glutamatergic signaling.^51^ Although our TRD subjects did not have overall decreased levels of FGF-2 compared to HC, those with the lowest levels of FGF-2 were more likely to exhibit positive treatment responses to ketamine. It is possible that patients with low levels of FGF-2 may represent a specific subset of patients who may be more likely to respond to ketamine treatment.

### Comparisons to recent literature and limitations

Our results suggest that ketamine treatment response is not directly related to a reduction of inflammatory cytokines such as IL-6. This runs counter to a recent smaller study, suggesting that decreases in IL-6 after ketamine treatment were predictive of antidepressant treatment response.^21^ That study by Yang *et al.* showed that patients who would go on to be ketamine responders had elevated baseline levels of IL-6, and that the levels decreased and remained low following ketamine treatment. Those who were depressed, but did not respond to ketamine in that study, had levels of IL-6 indistinguishable from healthy subjects. The study contained minimal information about the patient sample other than that they met criteria for MDD, so the comparability of the study samples is difficult to determine. The values of IL-6 reported in that study appeared to be noticeably elevated compared to what is typically observed. The HCs in Yang *et al.* were reported to have serum IL-6 levels of ~40 pg ml^−1^ and MDD patients ~80 pg ml^−1^ (our study, HC=0.83 pg ml^−1^; TRD=2.42 pg ml^−1^). In population level studies, it has been demonstrated that in healthy adults the median serum level of IL-6 is ~1.5 pg ml^−1^, with levels >10 pg ml^−1^ being unusual.^52^ The precise reason for the discrepant findings between the two studies remains unclear.

Another study examined the effect of ketamine on a smaller panel of cytokines.^22^ This study by Park *et al.* examined a group of patients with active MDD or bipolar depression. They found that treatment with ketamine resulted in a small and transient increase in IL-6 at 4 h after ketamine treatment, a change that did not correlate with treatment response. Although the finding is in the opposite direction, it is important to note that our studies agree that the effect of ketamine on peripheral IL-6 is minimal, transient and does not correlate with antidepressant response. The only other discrepancy between the Park *et al.* study and ours is that they do not see any alteration of IL-8 levels at 24 h after ketamine infusion. Given that this finding was not correlated with treatment response in our study, it is unlikely to be of clinical significance.

An additional important consideration is that, although our data showed altered cytokine levels in patients with TRD compared to HC, we cannot say that these changes are specific to TRD and would not be seen in treatment-responsive MDD. Although some studies have demonstrated elevated levels of cytokines in patients who are treatment-resistant to multiple trials of monoaminergic antidepressants,^3, 4, 8^ large meta-analyses of depressed patients not screened for treatment resistance also demonstrate elevated levels of inflammatory markers.^30^ Further study is needed to fully establish how cytokines may be altered specifically in treatment resistance and how this can be harnessed to better treat our patients. In addition, as with all studies examining the role of peripheral inflammation, the changes in peripheral cytokines that are seen here do not necessarily reflect the inflammatory state of the CNS. Further studies utilizing cerebrospinal fluid, post-mortem tissue and animal models will be necessary to fully characterize how ketamine may or may not affect expression of central inflammatory markers.

Overall, our study adds to the growing literature implicating dysregulation of immune signaling in depression. The nature of this dysregulation remains to be fully elicidated. We found that ketamine transiently lowers markers of inflammation, however these changes appear not to be directly linked to clinical antidepressant effects. Future studies with large sample sizes in well-characterized populations will be necessary to draw more definitive conclusions regarding the role of inflammation in treatment-resistant depression.

## Figures and Tables

**Table 1:** Demographic characteristics of HC and TRD populations

	*HC*	*TRD*	P*-value*
Paricipants, *n* (%)	26 (44%)	33 (56%)	
Gender (M/F)	14/14	21/14	0.303
Age at enrollment	39.0±11.1	44.8±13.6	0.082
Body mass index	27.2±5.3	28.2±6.2	0.189

*Race,* n *(%)*			0.062
Caucasian	12 (46%)	25 (76%)	
African-American	8 (31%)	4 (12%)	
Other	6 (23%)	4 (12%)	

*Ethnicity*			0.390
Hispanic	4 (14%)	2 (6%)	
Non-Hispanic	22 (85%)	31 (94%)	

*Depression history*			
# Lifetime MDE	—	4.71±7.3	
# Failed AD trials	—	5.21±2.7	
Failed SSRI, *n* (%)	—	28 (80%)	
Failed SNRI, *n* (%)	—	23 (66%)	
Failed atypical AD, *n* (%)	—	21 (60%)	
Failed MAOI, *n* (%)	—	8 (23%)	
Failed tricyclic, *n* (%)	—	7 (20%)	
Failed ECT, *n* (%)	—	5 (14%)	
Hx of augmentation trial, *n* (%)	—	18 (51%)	
Hx of suicide attempt	—	8 (23%)	
Past SUD, *n* (%)	—	2 (6%)	
Hx anxiety disorder, *n* (%)	—	7 (20%)	
Baseline MADRS score	—	33.0±4.4	
Baseline QIDS-SR score	3.5±3.5	17.9±4.4	<0.0001

Abbreviations: AD, antidepressant; ECT, electroconvulsive therapy; HC, healthy control; MADRS, Montgomery–Åsberg Depression Rating Scale; MAOI, monoamine oxidase inhibitor; MDE, major depressive episode; QIDS-SR, Quick Inventory of Depressive Symptomatology - Self Report; SNRI, serotonin norepinephrine reuptake inhibitor; SSRI, selective serotonin reuptake inhibitor; SUD, substance use disorder; TRD, treatment-resistant depression.

Anxiety disorder is defined as diagnosis of generalized anxiety disorder or panic disorder. Differences in categorical variables were analyzed with *χ*^2^ analysis, and continuous variables compared with *t*-test.

**Table 2:** Values of cytokines, chemokines and growth factors from HC and TRD subjects

	*HC*	*TRD*	*Mean difference*	P*-value*
	*Mean pg ml^−1^ (s.e.m.)*	*Mean pg ml^−1^ (s.e.m.)*		
IL-6	5.30 (2.07)	6.99 (2.45)	1.69	0.004**
IL-1α	66.57 (25.19)	196.79 (65.96)	130.23	0.083
IL-1β	1.54 (0.28)	5.69 (2.58)	4.15	0.119
TNF-α	5.67 (0.97)	10.71 (4.43)	5.04	0.45
EGF	75.83 (15.98)	99.80 (12.85)	23.97	0.237
Eotaxin	108.85 (9.92)	130.07 (9.20)	21.22	0.107
FGF-2	36.14 (8.35)	54.28 (12.05)	18.14	0.549
FLT3L	7.48 (3.42)	18.79 (8.72)	11.31	0.466
Fractalkine	40.39 (15.18)	136.08 (38.69)	95.69	0.073
G-CSF	33.08 (14.68)	45.52 (12.05)	12.43	0.037*
GM-CSF	13.40 (5.48)	44.66 (12.13)	31.26	0.02*
GRO	4456.84 (1145.12)	2034.05 (558.59)	−2422.79	0.153
IFN2a	22.24 (7.74)	59.71 (29.79	37.47	0.098
IFNr	43.60 (10.82)	81.43 (24.47)	37.83	0.315
IL-10	5.98 (3.08)	9.68 (5.39)	3.70	0.462
IL-12P40	53.50 (30.33)	40.81 (14.03)	−12.69	0.327
IL-12P70	4.92 (1.96)	10.92 (6.21)	6.00	0.144
IL-13	86.56 (37.40)	102.34 (32.64)	15.78	0.424
IL-15	6.27 (2.31)	8.74 (2.71)	2.47	0.559
IL-17a	11.09 (2.79)	20.51 (6.14)	9.43	0.329
Il-1ra	109.80 (53.92)	139.40 (41.91)	29.60	0.089
IL-2	3.78 (1.21)	6.80 (2.40)	3.02	0.331
IL-3	1.08 (0.08)	1.09 (0.08)	0.01	0.963
IL-4	3.14 (1.05)	10.86 (4.43)	7.71	0.199
IL-5	10.66 (4.54)	12.16 (4.06)	1.50	0.678
IL-7	5.68 (1.65)	6.48 (1.48)	0.81	0.716
IL-8	25.61 (8.15)	38.75 (8.77)	13.13	0.236
IL-9	2.76 (2.76)	3.83 (0.98)	1.07	0.316
IP-10	211.84 (21.32)	220.81 (20.05)	8.96	0.775
MCP-1	499.79 (53.26)	653.34 (58.29)	153.55	0.023*
MCP-3	72.23 (28.07)	102.22 (30.73)	29.99	0.484
MDC	835.68 (46.30)	950.18 (58.70)	114.50	0.71
Mip-1a	43.83 (29.20)	41.97 (22.64)	−1.86	0.43
Mip-1b	42.30 (12.11)	59.41 (12.73)	17.11	0.154
PDGF-AA	6384.27 (488.03)	7016.83 (424.69)	632.56	0.475
PDGF-BB	9524.58 (632.18)	11599.79 (454.07)	2075.21	0.005**
RANTES	2044.88 (204.00)	1892.45 (160.82)	−152.43	0.527
scd40L	2900.64 (411.10)	3730.59 (393.14)	829.95	0.088
TGF-α	3.73 (1.19)	10.05 (4.71)	6.31	0.175
TNF-β	155.66 (70.85)	207.64 (70.80)	51.98	0.434
VEGF	209.76 (41.59)	289.16 (50.67)	79.40	0.086

Abbreviations: CSF, cerebrospinal fluid; FGF-2, fibroblast growth factor 2; HC, healthy control; IL, interleukin; TRD, treatment-resistant depression.

All concentrations are presented as pg ml^−1^. Mean difference is calculated as raw difference of means with a positive value indicating higher levels in TRD (TRD–HC). *P-*values are derived from regression analyses of log-transformed data. *P<0.05; **P<0.01.
