# Supplementary material for: Altered peripheral immune profiles in treatment-resistant depression: response to ketamine and prediction of treatment outcome
Source: Transl Psychiatry. 2017 Mar 21;7(3):e1065–. doi: 10.1038/tp.2017.31 (PMC5416674; doi:10.1038/tp.2017.31)
Supplement: Supplementary Table S1 [file tp201731x1.pdf]

	Plate 1				Plate 2				Plate 3			
	R^2	CV	MinDC (pg/ml)	MaxDC (pg/ml)	R^2	CV	MinDC	MaxDC	R^2	CV	MinDC	MaxDC
EGF	1	1.08%	3.32	15524	1	1.20%	2.04	16284	1	1.06%	3.53	16223
EOTAXIN	0.999	2.04%	3.21	15884	1	1.80%	2.61	17050	1	1.89%	3.31	17709
FGF-2	1	1.03%	3.84	10521	0.999	2.35%	2.98	10182	0.999	1.50%	2.95	15239
FLT-3L	1	0.80%	3.58	16179	1	0.44%	3.18	16460	1	0.11%	1.78	20738
Fractakine	1	0.74%	3.29	20351	0.999	3.30%	2.63	10085	0.999	2.62%	2.19	11777
G-CSF	1	0.35%	2.76	16548	1	0.37%	2.75	16643	1	0.51%	2.8	18633
GM-CSF	1	1.14%	2.95	17198	1	0.65%	2.7	18241	1	0.53%	2.84	12108
GRO	1	0.40%	2.74	15070	1	0.82%	1.99	14355	1	0.54%	3.35	14850
IFNa2	1	0.81%	3.38	16520	1	0.031%	4.9	11515	1	1.48%	2.85	12930
IFNr	1	1.32%	2.73	18213	1	0.89%	2.57	18532	1	0.77%	2.68	17019
IL-10	0.999	1.84%	2.39	14980	1	0.88%	1.98	13173	1	0.72%	2.91	9557
IL-12P40	1	0.66%	3.22	15931	1	0.15%	1.89	14583	1	0.14%	1.28	19088
IL-12P70	1	1.52%	2.68	12790	1	0.79%	3	12575	1	1.44%	2.64	13910
IL-13	1	1.48%	2.83	16413	1	0.87%	2.74	11982	1	0.44%	2.96	13687
IL-15	1	1.12%	2.43	15987	1	0.39%	1.31	18473	1	0.50%	2.03	20038
IL-17A	1	1.65%	2.86	18417	1	1.00%	1.83	15481	1	0.75%	2.9	18969
IL-1a	1	0.49%	2.56	9945	0.999	1.02%	2.1	9811	0.999	2.28%	2.43	12389
IL-1b	1	1.57%	2.95	14692	1	1.62%	1.95	16391	1	1.39%	1.19	11942
IL-1RA	1	0.74%	2.87	16976	1	0.66%	2.14	17191	1	0.51%	2.65	15904
IL-2	1	1.01%	2.81	17867	1	0.43%	1.94	18475	1	0.58%	2.4	17502
IL-3	1	0.24%	1.53	29466	1	1.21%	2.45	18794	1	1.56%	2.07	11779
IL-4	1	1.54%	2.65	16737	1	1.49%	2.31	16213	1	0.95%	2.76	14522
IL-5	0.999	1.69%	3.07	20015	1	1.24%	2.14	8525	1	0.87%	2.95	11870
IL-6	1	0.077%	0.028	14664	0.999	2.94%	1.39	14168	0.999	2.59%	1.78	11622
IL-7	1	0.16%	2.1	11837	1	0.17%	3.99	11856	0.999	1.34%	1.42	11897
IL-8	1	1.13%	2.97	13012	1	1.28%	1.9	15181	1	0.32%	3.02	14628
IL-9	0.999	1.89%	2.81	18189	1	1.24%	3	12071	1	0.17%	0.98	20593
IP-10	0.999	0.054%	4.19	11275	1	1.39%	1.89	17752	0.999	1.09%	2.82	15360
MCP-1	0.999	2.70%	3.36	12133	1	1.59%	3.11	14586	1	1.54%	3.28	14520
MCP-3	0.999	0.095%	8.03	11683	0.999	2.84%	2.14	16871	1	0.28%	3.18	14923
MDC	1	0.034%	4.36	10964	0.999	2.59%	2.31	10847	0.994	0.14%	4.83	11673
MIP-1a	1	0.62%	5.13	7359	0.993	7.09%	1.84	8412	0.994	0.40%	5.21	10890
MIP-1b	1	0.30%	1.9	21598	1	0.29%	1.8	18900	1	0.39%	2.2	20577
PDGF-AA	1	0.78%	3.27	17279	1	0.29%	1.8	20676	1	0.36%	2.62	21051
PDGF-BB	1	0.14%	3.71	14317	1	0.21%	1.97	16018	1	0.55%	3.52	12793
RANTES	0.999	0.37%	13.42	18862	0.999	0.37%	1.7	19560	0.999	1.18%	3.04	14744
sCD40L	1	1.43%	16	17680	1	1.62%	2.86	17935	1	0.11%	0.7	42719
TGF-a	1	0.11%	0.86	7394	1	0.67%	1.96	9374	0.998	0.92%	4.34	11822
TNFa	0.999	2.16%	2.92	17111	1	1.63%	1.76	12968	1	1.05%	1.59	12016
TNFb	1	0.22%	0.83	39684	1	0.12%	0.93	21271	1	0.34%	2.54	21092
VEGF	0.999	2.55%	2.01	12202	0.999	3.95%	3.14	14625	1	1.27%	2.5	15062
