# Supplementary material for: Altered peripheral immune profiles in treatment-resistant depression: response to ketamine and prediction of treatment outcome
Source: Transl Psychiatry. 2017 Mar 21;7(3):e1065–. doi: 10.1038/tp.2017.31 (PMC5416674; doi:10.1038/tp.2017.31)
Supplement: Supplementary Table S2 [file tp201731x2.docx]

|  | ***Unadjusted P-value*** | | ***P-value adjusted for covariates*** | |
| --- | --- | --- | --- | --- |
| **IL-6** | **0.004**** | **0.017*** | |  |
| **IL-1α** | **0.083** | **0.167** | |  |
| **IL-1β** | **0.119** | **0.193** | |  |
| **TNF-α** | **0.45** | **0.947** | |  |
| **EGF** | **0.237** | **0.400** | |  |
| **Eotaxin** | **0.107** | **0.281** | |  |
| **FGF-2** | **0.549** | **0.633** | |  |
| **FLT3L** | **0.466** | **0.729** | |  |
| **Fractalkine** | **0.073** | **0.247** | |  |
| **G-CSF** | **0.037*** | **0.045*** | |  |
| **GM-CSF** | **0.02*** | **0.026*** | |  |
| **GRO** | **0.153** | **0.062** | |  |
| **IFN2a** | **0.098** | **0.115** | |  |
| **IFNr** | **0.315** | **0.147** | |  |
| **IL-10** | **0.462** | **0.541** | |  |
| **IL-12P40** | **0.327** | **0.427** | |  |
| **IL-12P70** | **0.144** | **0.195** | |  |
| **IL-13** | **0.424** | **0.440** | |  |
| **IL-15** | **0.559** | **0.539** | |  |
| **IL-17a** | **0.329** | **0.235** | |  |
| **Il-1ra** | **0.089** | **0.085** | |  |
| **IL-2** | **0.331** | **0.327** | |  |
| **IL-3** | **0.963** | **0.961** | |  |
| **IL-4** | **0.199** | **0.143** | |  |
| **IL-5** | **0.678** | **0.995** | |  |
| **IL-7** | **0.716** | **0.575** | |  |
| **IL-8** | **0.236** | **0.299** | |  |
| **IL-9** | **0.316** | **0.282** | |  |
| **IP-10** | **0.775** | **0.918** | |  |
| **MCP-1** | **0.023*** | **0.045*** | |  |
| **MCP-3** | **0.484** | **0.431** | |  |
| **MDC** | **0.71** | **0.530** | |  |
| **Mip-1a** | **0.43** | **0.364** | |  |
| **Mip-1b** | **0.154** | **0.163** | |  |
| **PDGF-AA** | **0.475** | **0.848** | |  |
| **PDGF-BB** | **0.005**** | **0.014*** | |  |
| **RANTES** | **0.527** | **0.641** | |  |
| **scd40L** | **0.088** | **0.256** | |  |
| **TGF-α** | **0.175** | **0.354** | |  |
| **TNF-β** | **0.434** | **0.407** | |  |
| **VEGF** | **0.086** | **0.235** | |  |
