# Supplementary figures and images for: Altered peripheral immune profiles in treatment-resistant depression: response to ketamine and prediction of treatment outcome
Source: Transl Psychiatry. 2017 Mar 21;7(3):e1065–. doi: 10.1038/tp.2017.31 (PMC5416674; doi:10.1038/tp.2017.31)

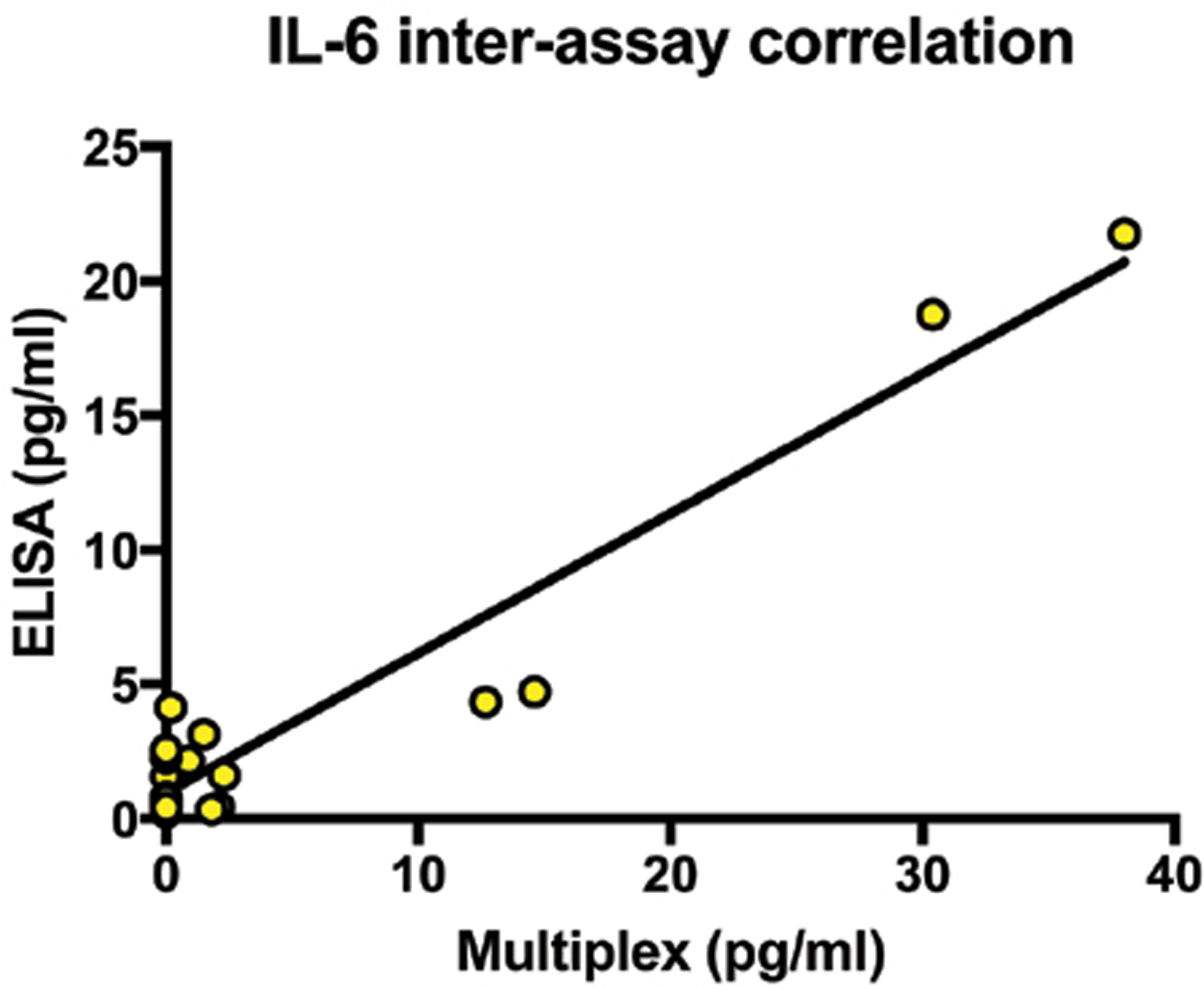

Supplement: Supplementary Figure S1 [file tp201731x3.tif]

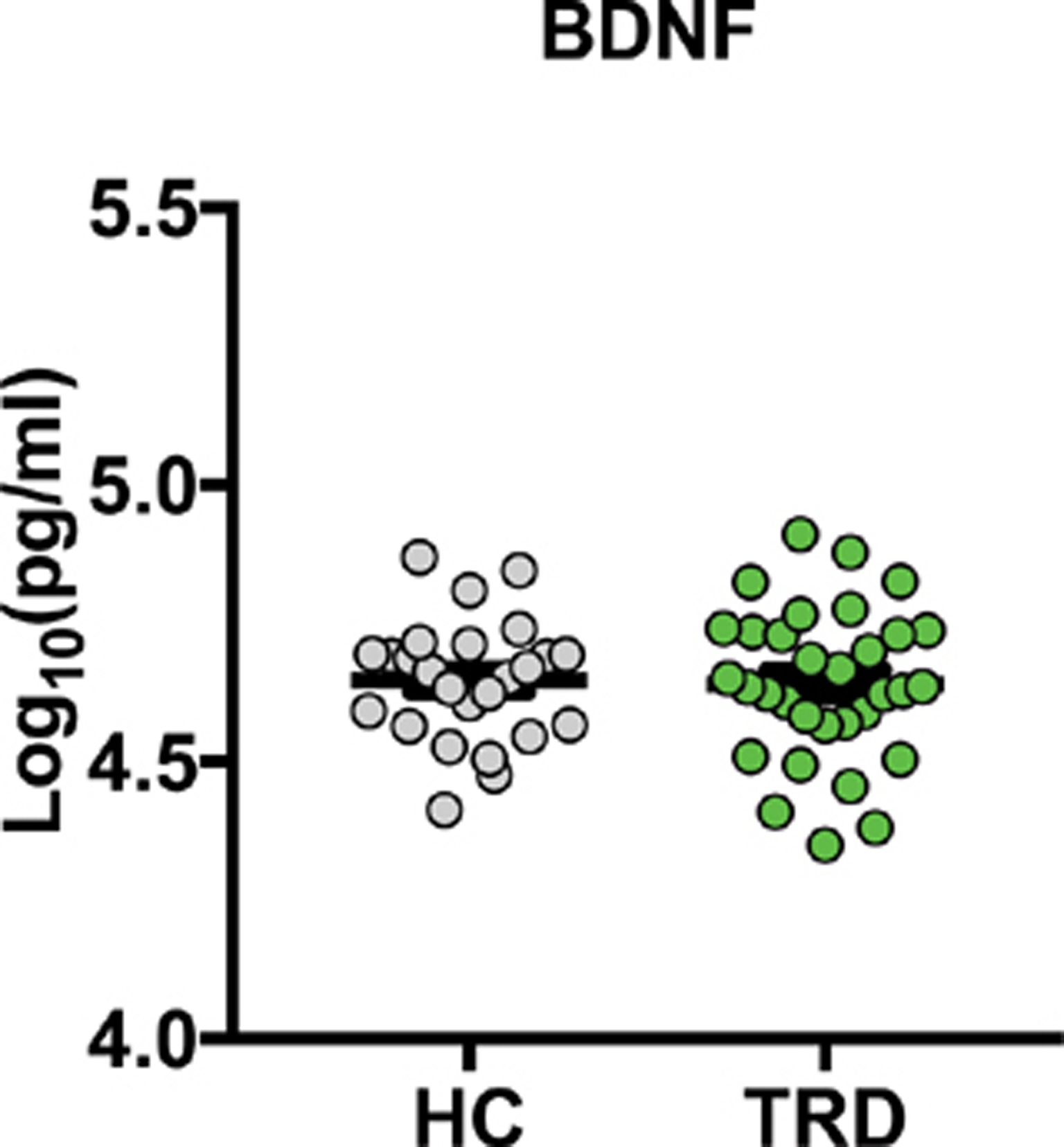

Supplement: Supplementary Figure S2 [file tp201731x4.tif]
