# Supplementary material for: Altered peripheral immune profiles in treatment-resistant depression: response to ketamine and prediction of treatment outcome
Source: Transl Psychiatry. 2017 Mar 21;7(3):e1065–. doi: 10.1038/tp.2017.31 (PMC5416674; doi:10.1038/tp.2017.31)
Supplement: Supplementary Figure and Table Legends [file tp201731x5.doc]

**Supplementary Tables**

**Table S1 –Quality control data from multiplex assay.** Samples for this assay were divided evenly between three plates from the same lot run at the same time. This table includes the R^2^ values for the standard curve, the coefficients of variation, and the minimum and maximum levels of detection for each analyte.

**Table S2 – P values adjusted for covariates.** For each analyte regression analysis was performed for TRD group only (left column), and with covariates of sex, age, BMI and race included (right column).

**Supplementary Figure**

**Figure S1** – Pg/ml levels of IL-6 were compared between the current multiplex assay and a more traditional ELISA assay on the same samples. The values showed strong correlation with a Pearson’s r of 0.957 (*p* = 0.0009)

**Figure S**2 – No detectable differences were seen in serum BDNF between HC and TRD at baseline (p = 0.86). Additionally, levels of BDNF did not change with ketamine treatment and were not predictive of antidepressant response to ketamine (data not shown).
